# Initial Psychometric Evaluation of the Social Safeness and Pleasure Scale Japanese Version

**DOI:** 10.3390/bs16061030

**Published:** 2026-06-19

**Authors:** Kenichi Asano, Asa Nagae, Yasuhiro Kotera, Rhea Takahashi, Jaskaran Basran, Paul Gilbert

**Affiliations:** 1 Department of Psychology, Institute of Human Sciences, University of Tsukuba, Tsukuba 305-0006, Japan; 2 Graduate School of Contemporary Psychology, Rikkyo University, Saitama 352-8558, Japan; 3 Faculty of Medicine and Health Sciences, University of Nottingham, Nottingham NG7 2TU, UK; 4 Center for Infectious Disease Education and Research, The University of Osaka, Suita 565-0871, Japan; 5 Department of Social Sciences, Azerbaijan University, Baku AZ1007, Azerbaijan; 6 Shinjuku-Yoyogi Mental Lab Clinic, Tokyo 151-0051, Japan; 7 Centre for Compassion Research and Training, College of Health, Psychology and Social Care, University of Derby, Derby DE22 1G, UK

**Keywords:** depression, mental health, social safeness, social support, well-being

## Abstract

This study conducted an initial psychometric evaluation of the Japanese version of the Social Safeness and Pleasure Scale (SSPS-J). In Study 1 (*N* = 477), exploratory factor analysis supported a single-factor structure with excellent internal consistency (alpha = 0.95, omega = 0.95). Significant correlations with depression (*r* = −0.53), anxiety (*r* = −0.26), stress (*r* = −0.36), life satisfaction (*r* = 0.67), and social support (*r* = 0.47–0.52) demonstrated robust convergent validity. In Study 2, confirmatory factor analysis (*N* = 262) confirmed the reproducibility of the single-factor model with an acceptable overall fit (CFI = 0.943, SRMR = 0.036, RMSEA = 0.108). Test–retest reliability over a three-week interval (*N* = 113) was also high (ICC = 0.88). These results suggest that the SSPS-J is a reliable and valid preliminary measure for assessing social safeness in the Japanese general population.

## 1. Introduction

The Social Safeness and Pleasure Scale (SSPS) was established to quantify experiences and feelings of social safeness (Gilbert et al., 2009). Social safeness differs from social safety. Safety is linked to the degree of presence or absence and potential harmfulness of a threat. It is monitored through the evolved threat system, utilizing the amygdala and sympathetic nervous system (Panksepp, 1988). In contrast, social safeness is monitored through signals of support and helpfulness utilizing the parasympathetic system, oxytocin and neural circuits that detect friendliness (Gilbert, 1993, 2000, 2020; J. J. Kim et al., 2020; Porges, 2021). This system evolved partly with the attachment system such that the parent acts as a secure base and safe haven for the infant and child (Cassidy & Shaver, 2016). When stressed, the infant receives inputs from the caregiver that stimulate the social safeness, vagus and oxytocin system (Gilbert, 2020). Importantly too, humans are a highly social species who are significantly psychophysiologically regulated through our relationships (Camilleri et al., 2023; Klimecki, 2015; Mikulincer & Shaver, 2014) especially friendships (Dunbar, 2022). Hence, social safeness is linked to the degree to which individuals feel they operate within groups and communities of friendliness, support and belonging. Emotions arising from social safeness can be distinguished from positive emotions of excitement and relaxation and are associated negatively with indices of mental health difficulties (Gilbert et al., 2009).

Gilbert et al. (2009) developed the Social Safeness and Pleasure Scale to distinguish social positive emotions from nonsocial ones. It has items such as “I feel connected to others; I have a sense of being cared about in the world; I feel a sense of belonging”. Findings from previous research on the multifaceted role of social safeness can be organized into three primary domains: intrapersonal factors, interpersonal dynamics, and psychological maladjustment.

Regarding intrapersonal factors and well-being, studies have demonstrated that social safeness is positively associated with self-concept and self-compassion (Alavi, 2021; Kelly & Dupasquier, 2016; Lefebvre et al., 2021; Matos et al., 2022; Nguyen et al., 2022; Satici et al., 2013; Uysal, 2015). It is also associated with psychological vulnerability that is mediated by life satisfaction (Satici et al., 2016) and both individual and group-level innovation (Lefebvre et al., 2021).

In terms of interpersonal dynamics, social safeness is rooted in relational experiences. Kelly and Dupasquier (2016) found that social safeness was positively correlated with parental warmth but negatively correlated with parental rejection and overprotection (Marta-Simões & Ferreira, 2020). Lower social safeness is also associated with interpersonal problems (Alavi et al., 2016), detachment, and mistrust (Isaksson et al., 2022). While social safeness overlaps with perceived social support, they are conceptually distinct; safeness reflects an internal psychophysiological state of feeling safe, whereas support focuses on the availability of external resources (Kelly et al., 2012; Nguyen et al., 2022). Additionally, social safeness has been found to mitigate the negative impact of interpersonal stressors, such as mobbing on work meaning (Saricam, 2016).

Finally, regarding psychological maladjustment, studies have demonstrated that social safeness is negatively correlated with depression, anxiety, and stress in patients and students across various cultures (Gilbert et al., 2009; McEwan et al., 2012; Alavi, 2021; Isaksson et al., 2022). Social safeness is also linked to problematic tendencies such as social phobia (Alavi et al., 2016), psychological stigma (Ozden & Deniz, 2018), and addictive technology use (Akin & Akin, 2015; Uysal, 2015). Importantly, low social safeness is significantly associated with clinical difficulties such as fears of compassion (for self, from others, and for others), hyper-competitiveness, and striving to avoid inferiority (Basran et al., 2019). A Swedish study further found that the SSPS was negatively correlated with mental health difficulties, especially for patients with eating disorders and borderline personality difficulties (Isaksson et al., 2022).

These findings highlight the relevance of social safeness as a clinical focus for mental health. Compassion-Focused Therapy or Compassionate Mind Training has been shown to increase social safeness (Cuppage et al., 2018; Gilbert & Procter, 2006; Mayhew & Gilbert, 2008). Interventions that build social safeness and social connectedness have been shown to have wide-ranging effects on health in those communities (Abel & Clarke, 2020).

Despite these beneficial aspects of social safeness, there are no reports from East Asian countries owing to a lack of validated measurement tools. In the Japanese context, longitudinal data from 1983 to 2023 indicate a gradual increase in feelings of loneliness, a trend that appears to have been further intensified by the COVID-19 pandemic (Homma & Takase, 2026). Moreover, Sugaya et al. (2024) reported that the challenges of loneliness and social isolation emerging during the pandemic have persisted. Furthermore, a comparative study involving 22 countries identified Japan as having a high prevalence of social isolation and, along with Turkey, a relatively low quality of social support (Pei & Zaki, 2025). As these observations are particularly notable among younger populations, there is a clear rationale for investigating the construct of social safeness within this demographic.

Previous validation studies of the SSPS have consistently demonstrated robust psychometric properties across various cultures. While all versions have reported high internal consistency, the methodologies for structural validation have varied: the English and Persian versions established a single-factor structure through exploratory factor analysis (Gilbert et al., 2009; Alavi et al., 2016), whereas the Swedish and Portuguese versions confirmed this unidimensionally using confirmatory factor analysis (Isaksson et al., 2022; Miguel et al., 2022). These findings underscore the cross-cultural stability of the SSPS’s internal structure and provide a strong foundation for its application in the Japanese context.

Therefore, our goal was to provide an initial validation of the Japanese version of the SSPS (SSPS-J). The primary aim of Study 1 was to prepare an initial draft of the SSPS-J and explore its factor structure, internal consistency, and convergent validity (examining the relationships between mental health, subjective well-being, and social support). Study 2 aimed to provide preliminary evidence regarding the reproducibility of the factor structure using confirmatory factor analysis and to verify the three-week test–retest reliability using intraclass correlation coefficients.

## 2. Study 1

### 2.1. Materials and Methods

#### 2.1.1. Participants and Procedure

Participants were recruited via an anonymous, individual-response Internet-based survey provided by the panel survey service of Questant. A total of 562 adults agreed to participate in the survey. After excluding invalid responses, 477 responses (233 males, 244 females; mean age, 45.00 years, SD = 14.00) were analyzed. Ethical approval was obtained from the Research Ethics Committee of Mejiro University in the Humanities and Social Sciences (Approval No. 20 person-014).

#### 2.1.2. Measures

The draft of the Japanese version of the SSPS (SSPS-J) was prepared following established guidelines (Beaton et al., 2000). Two bilingual psychologists independently translated the SSPS into Japanese, and the translations were then integrated through a meeting involving the first author (KA). Subsequently, the integrated Japanese version was independently back-translated by two psychologists via a translation company. The back-translations were reviewed for consistency with the original version by the original author (PG), and a pilot survey was conducted with eight graduate psychology students to confirm face validity.

The draft of the SSPS-J consists of 11 items, and participants were asked to choose the one how frequently they experience the item content on a five-point Likert scale ranging from 1 (almost never) to 5 (almost all the time). The Cronbach’s α coefficient of the original version was 0.91 (Gilbert et al., 2009).

Regarding psychological maladjustment, the original version of the DASS consisted of 21 items (Antony et al., 1998). Through verification of the 21-item version’s factorial validity, some items were excluded and finalized as the 15-item version (DASS-15). The DASS-15 consists of three five-item subscales (depression, anxiety, and stress) with high reliability and validity (Adachi & Ueno, 2011). Participants responded to items regarding their habits and experiences in the previous week using a Likert scale ranging from 0 (did not apply to me at all) to 3 (applied to me very much, or most of the time). The α coefficients of the three factors from the previous study were 0.76–0.86 (Adachi & Ueno, 2011). We employed the DASS-15 as an indicator of convergent validity and hypothesized that the SSPS-J has a negative correlation with the three subscales of the DASS-15. Based on previous reports, we hypothesized that the SSPS-J correlated with depression moderately, anxiety weakly, and stress weakly or moderately (Alavi, 2021; McEwan et al., 2012).

To evaluate intrapersonal factors, we employed the Satisfaction with Life Scale (SWLS) to measure subjective well-being. Previous research has shown good validity and reliability in both the English and Japanese versions (Diener et al., 1985). The Japanese version of the SWLS consists of five items, and participants responded on a Likert scale of 1 (did not apply to me at all) to 7 (applied to me very much, or most of the time). The α coefficient from a previous study was 0.84 (Sumino, 1994). We employed SWLS as an indicator of convergent validity and hypothesized that the SSPS-J has a positive correlation ranging from medium to large with SWLS, as shown in previous reports on subjective well-being (Lefebvre et al., 2021; Nguyen et al., 2022; Satici et al., 2016).

For interpersonal factors, we employed scales of emotional and instrumental social support from the Japanese version of the brief Coping Orientation to Problems Experienced (COPE) to measure emotional and instrumental social support. (Otsuka, 2008). The brief COPE is a short version of the COPE that measures coping strategies and is widely used in English; it has adequate reliability and validity (Carver, 1997; Carver et al., 1989). The Japanese version of the brief COPE also has sufficient reliability and validity and includes two items for each of the 14 factors, including the use of emotional and instrumental social support. The α coefficients reported in a previous study were 0.72–0.80 (Otsuka, 2008). SSPS items relate to feelings of belonging, acceptance, and warmth from others, and previous reports showed that SSPS correlates with received social support (Kelly & Dupasquier, 2016). Another study showed a large correlation with perceived social support (Kelly et al., 2012). While social safeness and social support are conceptually distinct, we hypothesized that the SSPS-J largely correlated with emotional social support and instrumental social support as convergent validity.

#### 2.1.3. Data Analysis

Data analysis was conducted using the free statistical software HAD17 (Shimizu, 2016). There were no missing data because the data collection was entrusted to a survey company.

To investigate the factor structure of the SSPS-J, we first performed parallel analysis and the Minimum Average Partial (MAP) test. Next, we conducted exploratory factor analysis (EFA) with the maximum likelihood method, based on the number of factors suggested by these criteria. The factor structure was verified based on factor loadings, and internal consistency was assessed using Cronbach’s *α* and McDonald’s *ω* coefficients.

The scores of the variables were summed for each factor following previous research, and Pearson correlation coefficients were calculated to verify convergent validity. Based on Cohen’s (1988) criteria, we interpreted the effect size *r*, ≥0.10 as small, ≥0.30 as medium, and ≥0.50 as large. Finally, an independent *t*-test was conducted to examine gender differences in SSPS-J scores.

Regarding sample size justification, a power analysis using G*Power 3.1 (Faul et al., 2007) indicated that a sample size of *N* = 343 is required to detect a small-to-medium effect size (*r* = 0.15) with a power of 0.80 and α = 0.05. Our obtained sample size (*N* = 477) comfortably exceeds this requirement, ensuring sufficient power to detect the hypothesized relationships and confirming the robustness of non-significant findings, such as the absence of gender differences.

### 2.2. Results

Descriptive statistics for the 11 items of the SSPS-J showed that skewness ranged from −0.36 to 0.11, and kurtosis ranged from −0.68 to −0.21. These values were well within the acceptable range (absolute skewness < 2 and kurtosis < 7; H. Y. Kim, 2013), justifying the use of maximum likelihood estimation in the subsequent factor analysis.

Parallel analysis and the MAP test suggested a single-factor structure. Specifically, the parallel analysis indicated that only the first observed eigenvalue (7.29) exceeded the corresponding random eigenvalue (1.26), while the second observed eigenvalue (0.83) was smaller than its random counterpart (1.18). Furthermore, the MAP test reached its minimum value at the first factor (MAP = 0.024), consistently supporting the unidimensionality of the scale. Accordingly, an exploratory factor analysis (EFA) was performed with a single-factor model (Table 1). All items showed sufficient factor loadings (ranging from 0.48 to 0.88), and we adopted a single-factor structure. Both Cronbach’s α and McDonald’s ω coefficients were 0.95.

Table 2 presents the descriptive statistics, Cronbach’s α coefficients, and correlation coefficients for all variables. As hypothesized, the SSPS-J was significantly correlated with depression, subjective well-being, and emotional social support, as well as anxiety, stress, and instrumental social support. Regarding gender differences, an independent two-tailed *t*-test revealed no significant difference in SSPS-J scores (*t* (475) = −0.21, *p* = 0.83).

## 3. Study 2

### 3.1. Materials and Methods

#### 3.1.1. Participants and Procedure

Participants were recruited via an anonymous, individual-response Internet-based survey provided by Freeasy’s panel survey service. In total, 300 adults agreed to participate in the survey. After excluding invalid responses, 262 participants (125 males, 137 females; mean age, 41.07 years, SD = 16.25) were included in the confirmatory factor analysis (CFA) at Time 1.

At Time 2 (three weeks later), 167 of the original participants responded. To ensure a stable sample for assessing test–retest reliability, we selected participants who reported their condition as “unchanged”, “slightly improved”, or “slightly worse” on a global rating of change scale. This resulted in a final sample of 113 participants (55 males, 58 females; mean age, 45.02 years, SD = 15.37) for the calculation of the intraclass correlation coefficient (ICC).

Ethical approval was obtained from the Research Ethics Committee of Mejiro University in the Humanities and Social Sciences (Approval No. 22 person-031).

#### 3.1.2. Measures

The SSPS-J developed in Study 1 was employed.

Additionally, a global rating of change (GRC) assessment was used to assess stability for test–retest reliability. Participants rated the extent to which their mental and physical state had changed since Time 1 on a scale of 1 (very much improvement) to 7 (very much deterioration). Only those reporting minimal change (scores of 3, 4 or 5) were included in the ICC calculation.

#### 3.1.3. Data Analysis

Data analysis was conducted using HAD (Shimizu, 2016). There were no missing data. CFA was employed to verify the reproducibility of the factor structure using the maximum likelihood method. The ICC (two-way mixed effects model, absolute agreement; ICC(2,1)) was calculated to evaluate test–retest reliability.

The sample size for Study 2 (*N* = 262) was determined to be sufficient for evaluating the structural stability and reliability of the scale. This size exceeds the common recommendation of at least 10 participants per item for confirmatory factor analysis (Bentler & Chou, 1987) and is substantially larger than the sample sizes used in previous SSPS validation studies (e.g., Alavi et al., 2016; Isaksson et al., 2022). Furthermore, this sample size provides high precision for estimating test–retest reliability using intraclass correlation coefficients.

### 3.2. Results

To test the reproducibility of the factor structure suggested in Study 1, a CFA was conducted (Table 3). All path coefficients were significant, and the goodness-of-fit indices were as follows: χ^2^(44) = 179.323, *p* < 0.001, CFI = 0.943, RMSEA = 0.108 (90% CI [0.092, 0.125]), SRMR = 0.036, and AIC = 223.323. Although the RMSEA exceeded the conventional threshold, the model was interpreted as having an acceptable fit for an initial validation, given the model’s parsimony and the absence of significant standardized residuals and strong factor loadings (MacCallum et al., 1996).

In Study 2, the SSPS-J showed mean scores of 31.08 (SD = 10.03) at Time 1 and 32.35 (SD = 10.74) at Time 2. Internal consistency remained excellent at both time points (Time 1: α = 0.95, ω = 0.95; Time 2: α = 0.95, ω = 0.95), and the ICC was 0.88 (95% CI [0.83, 0.92]).

## 4. Discussion

We developed a draft of the Japanese version of the SSPS with content validity, explored its factor structure, and verified its structural validity, internal consistency, convergent validity, and test–retest reliability.

### 4.1. Content Validity and Structural Validity

We conducted reliable procedures based on translation guidelines for cross-cultural adaptation (Beaton et al., 2000). We also conducted a pilot survey to assess face validity. These steps ensured the same content validity as the original version. Results from exploratory factor analysis revealed a structure similar to the original version, which was reproduced in confirmatory factor analysis with sufficient goodness-of-fit indicators. Therefore, we can conclude that the SSPS-J has good structural validity. Our findings are highly consistent with the psychometric properties reported in previous validation studies; specifically, the unidimensionality we observed aligns with the EFA results of the English and Persian versions (Gilbert et al., 2009; Alavi et al., 2016) and the CFA results of the Swedish and Portuguese versions (Isaksson et al., 2022; Miguel et al., 2022).

Regarding the model fit in Study 2, the RMSEA (0.108) exceeded the ideal threshold of 0.08, although other indices such as the CFI (0.943) and SRMR (0.036) demonstrated an acceptable fit. This discrepancy is often observed in models with low degrees of freedom and parsimonious structures, where RMSEA tends to over-reject true models. The marginal RMSEA in the SSPS-J may also be influenced by the relatively low factor loading of Item 4 (0.482 in Study 1), a pattern occasionally noted in previous studies. Despite this, the absence of significant standardized residuals and strong factor loadings supports the structural validity of the single-factor model. Furthermore, no significant gender differences were observed, suggesting the SSPS-J assesses psychological tendencies independent of gender.

### 4.2. Internal Consistency and Test–Retest Reliability

The α and ω coefficients were high (0.95), indicating excellent internal consistency. The test–retest reliability was also high over a 3-week interval (ICC = 0.88). While test–retest reliability for the original version has not been reported, our results are consistent with the high reliability found in the Swedish (*r* = 0.92) and Persian (*r* = 0.82) versions (Alavi et al., 2016; Isaksson et al., 2022). Thus, the SSPS-J is a stable and reliable measure for the Japanese population.

### 4.3. Convergent Validity

As hypothesized, the convergent validity of the SSPS-J was supported across the three domains: psychological maladjustment, intrapersonal factors, and interpersonal dynamics.

Regarding psychological maladjustment, the SSPS-J showed significant negative correlations with depression, anxiety, and stress. These results align with previous studies, suggesting that social safeness acts as a protective factor against psychological distress (Alavi, 2021; McEwan et al., 2012). Notably, low social safeness is often associated with clinical difficulties such as fears of compassion (Basran et al., 2019), and the SSPS-J is expected to be a key indicator in understanding these vulnerabilities.

In terms of intrapersonal factors, the SSPS-J showed a positive correlation with subjective well-being (life satisfaction), as hypothesized (Satici et al., 2016). Social safeness is theoretically rooted in a positively related affect system (e.g., endorphins and oxytocin) that facilitates soothing and contentment (Gilbert et al., 2009). Our findings provide empirical support for this link within the Japanese context.

Finally, regarding interpersonal dynamics, the SSPS-J correlated moderately to strongly with emotional social support and moderately with instrumental social support. While social safeness overlaps with perceived social support, it specifically captures the internal psychophysiological state of feeling safe and connected, rather than just the availability of external resources (Kelly et al., 2012). The consistent correlations across these domains confirm the sufficient convergent validity of the SSPS-J.

### 4.4. Limitations and Future Research Suggestions

This study utilized a sample size that exceeds that of several previous reports on the development of the SSPS (N = 477 in Study 1 and N = 262 in Study 2), featuring a wide age range and balanced gender distribution. By surpassing the sample scales of earlier validation studies (e.g., Alavi et al., 2016; Isaksson et al., 2022; Satici et al., 2016), our findings provide relatively robust evidence for the psychometric properties of the SSPS-J in the Japanese general population. However, some limitations should be noted.

First, data collection was conducted via an online survey agency, which may cause recruitment bias compared to voluntary participation.

Second, this study focused on a general sample. Considering the importance of social safeness in clinical contexts, future research is required to verify the psychometric properties in clinical samples, such as individuals with eating disorders or personality difficulties (Isaksson et al., 2022).

Third, future studies could employ item response theory (IRT) to clarify item difficulty and discrimination for more precise measurement. Furthermore, future research should examine the incremental validity of the SSPS-J to determine if it predicts mental health outcomes above and beyond existing social support measures. Additionally, establishing measurement invariance across genders and clinical groups will be a critical step in further demonstrating the scale’s robustness and cross-cultural applicability.

Fourth, while the RMSEA in Study 2 was above the ideal threshold, further refinement of the scale and testing its cross-cultural invariance in even larger and more diverse samples may help clarify its structural robustness.

Despite these limitations, the SSPS-J serves as a preliminary yet robust tool for accumulating evidence on social safeness and its role in mental health and interpersonal relationships in Japan.

## 5. Conclusions

This study provided the initial validation of the Japanese version of the SSPS (SSPS-J). The results demonstrated sufficient reliability and validity within the Japanese general population. Specifically, the SSPS-J showed robust convergent validity across three domains: psychological maladjustment, intrapersonal factors (subjective well-being), and interpersonal dynamics (social support). These findings suggest that the SSPS-J is a useful and stable preliminary measure for assessing social safeness, providing a valuable foundation for future research and clinical practice in Japan.

## Figures and Tables

**Table 1:** Factor loadings of SSPS-J on EFA.

No.	Item	Loadings
1	I feel content within my relationships.	0.769
2	I feel easily soothed by those around me.	0.784
3	I feel connected to others.	0.813
4	I feel part of something greater than myself.	0.482
5	I have a sense of being cared about in the world.	0.773
6	I feel secure and wanted.	0.827
7	I feel a sense of belonging.	0.835
8	I feel accepted by people.	0.883
9	I feel understood by people.	0.876
10	I feel a sense of warmth in my relationships with people.	0.848
11	I find it easy to feel calmed by people close to me.	0.773

**Table 2:** Descriptive statistics and correlation matrix in Study 1 (*N* = 477).

	Variable	Mean	SD	α	1	2	3	4	5	6	7
1	SSPS-J	32.87	9.05	0.95	-	−0.53 **	−0.26 **	−0.36 **	0.67 **	0.52 **	0.47 **
						[−0.59, −0.46]	[−0.34, −0.17]	[−0.43, −0.27]	[0.62, 0.72]	[0.45, 0.58]	[0.40, 0.54]
2	Depression	8.93	3.79	0.91		-	0.74 **	0.75 **	−0.39 **	−0.24 **	−0.19 **
							[0.69, 0.77]	[0.70, 0.78]	[−0.47, −0.32]	[−0.32, −0.25]	[−0.27, −0.10]
3	Anxiety	7.49	3.33	0.91			-	0.82 **	−0.12 **	−0.07	0.00
								[0.79, 0.85]	[−0.21, −0.04]	[−0.15, 0.03]	[−0.09, 0.09]
4	Stress	8.63	3.45	0.88				-	−0.23 **	−0.03	−0.02
									[−0.32, −0.15]	[−0.12, 0.06]	[−0.07, 0.11]
5	SWLS	17.56	6.80	0.93					-	0.36 **	0.30 **
										[0.28, 0.44]	[0.22, 0.38]
6	Emotional	4.95	1.41	0.88						-	0.78 **
	Social Support										[0.75, 0.82]
7	Instrumental	4.92	1.46	0.89							-
	Social Support										

Note. Brackets indicate 95% confidence intervals. ** *p* < 0.01.

**Table 3:** Factor loadings of SSPS-J on CFA.

No.	Loadings [95%CI]
1	0.82 [0.70, 0.94]
2	0.90 [0.78, 1.01]
3	0.96 [0.85, 1.07]
4	0.74 [0.62, 0.86]
5	0.86 [0.75, 0.96]
6	0.90 [0.79, 1.00]
7	0.91 [0.79, 1.04]
8	0.98 [0.87, 1.09]
9	0.94 [0.83, 1.05]
10	0.93 [0.81, 1.04]
11	0.85 [0.74, 0.96]

## Data Availability

The data presented in this study are available on request from the corresponding author. The data are not publicly available due to privacy and ethical restrictions.

## Abbreviations

The following abbreviations are used in this manuscript:

SSPS-J	Social Safeness and Pleasure Scale Japanese Version
SSPS	Social Safeness and Pleasure Scale
DASS-15	Depression, Anxiety, and Stress Scale-15
SWLS	Satisfaction with Life Scale
COPE	Coping Orientation to Problems Experienced
